# Probe ultrasonification of egg yolk plasma forms low-density lipoprotein nanoparticles that efficiently protect canine semen during cryofreezing

**DOI:** 10.1016/j.jbc.2022.101975

**Published:** 2022-04-28

**Authors:** Edenara Anastácio da Silva, Carine Dahl Corcini, Francisco de Assis Araújo Camelo Junior, Diego Martins, Stela Mari Meneghello Gheller, Gabriela Hädrich, Cristiana Lima Dora, Antonio Sergio Varela Junior

**Affiliations:** 1 Faculdade de Veterinária, Universidade Federal de Pelotas, Capão do Leão, Brazil; 2 Department of Pharmaceutical Technology and Biopharmacy, University of Vienna, Vienna, Austria; 3 Laboratório de Nanotecnologia Aplicada à Saúde, Universidade Federal do Rio Grande, Rio Grande, Brazil; 4 Reprodução Animal Comparada, Instituto de Ciências Biológicas, Universidade Federal do Rio Grande, Rio Grande, Brazil

**Keywords:** ACROs, acrosomes, BCF, beat cross frequency, CFDA, carboxyfluorescein diacetate, EYP, egg yolk plasma, LDL, low-density lipoprotein, LPO, lipid peroxidation, MF, membrane fluidity, MI, membrane integrity, PDI, polydispersion index, PM, progressive motility, ROS, reactive oxygen species

## Abstract

Around the world, many couples have turned to *in vitro* fertilization as a viable solution to fertility issues. Low-density lipoprotein (LDL) is a protein best known for transporting fat molecules throughout the body, but it has also been shown to protect sperm cells during cryopreservation due to its micellar structure. In the present study, we aimed to evaluate different protocols for the preparation of nanoparticles from egg yolk plasma (EYP) containing LDL to improve the viability of cryopreserved canine semen. EYP was subjected to three distinct treatments: ultrasonification in an ultrasound bath at 40 kHz for 30 min (LDL-B); ultrasonification *via* an ultrasound probe at 50% amplitude for 30 min (LDL-P); and high-pressure homogenization at 10,000 PSI for six cycles (LDL-H). Sperm quality was assessed after thawing using computer-assisted sperm analysis and flow cytometry. The results revealed that compared to the EYP control, the LDL-P formulation presented significantly higher efficiency (*p* < 0.05) in maintaining total and progressive sperm motility, sperm membrane integrity and fluidity, and levels of intracellular reactive oxygen species. The LDL-P nanoparticles had an average size of approximately 250 nm, a PDI value of 0.3, and −1.15 mV of zeta potential, which are very important because it is an indicator of the stability of a colloidal dispersion. Therefore, we conclude that ultrasonication of EYP using a probe is an efficient method for the preparation of LDL nanoparticles that would enhance the cryoprotection of semen during freezing.

Nanoscale structures exhibit functional properties that are unique and not observed at the macroscale (1). This is mainly due to the high surface area at the nano-level, which allows an intense interaction of the nanoparticles with the matrix they are introduced in (2). In animal reproduction, certain metal-conjugated nanoparticles (3), natural extracts (4), and vitamin E nanoemulsions (5) with antioxidant properties are reported to be among the most promising candidates for application in sperm cryopreservation (6).

Low-density lipoproteins (LDLs) at a concentration of 8% have been applied in canine semen freezing with encouraging results (2). However, the traditional process of LDL extraction was multi-step, and mistakes were inevitable during the procedure. Therefore, in 2016, studies on simplified LDL extraction processes were published, and it were also reported that egg yolk plasma (EYP) exhibited similar properties for canine semen cryopreservation (7, 8). EYP offers several advantages, including easier procurement, rapid and inexpensive extraction directly from the entire yolk, and the possibility of sterilization and upscaling to the industrial level (9).

Belala and Delay (10) experimentally demonstrated the equivalence of LDL and liposomes in the cryoprotection of dog sperm. However, in another study on the biomimetic membrane, differences were observed between the interaction of liposomes and that of LDL with the sperm membrane and prostatic fluid, which emphasized the unique functional properties exhibited by LDL lipids during cryopreservation.

LDL comprises a spherical molecule, with the size of its monomers ranging from 8 to 1200 nm and a lipid core of triglycerides and cholesterol esters surrounded by a film of phospholipids and proteins (11, 12, 13, 14). When producing particles at nanometric scales, the methodologies used for the synthesis influence the homogenization, reduction, dispersion, and emulsification of the synthesized compounds (15). Ultrasonic generators and high-pressure homogenizers are the mechanical methods of energy formation that may be used for synthesizing nanostructures composed of lipids (16). Therefore, it is possible to manipulate the nanoforms of LDL micelles derived from EYP, with possible increases in the efficiency of interaction and absorption during cryopreservation. In this context, the present study aimed to evaluate different protocols for the preparation of LDL nanoparticles from EYP containing 8% LDL for the maintenance of the viability of cryopreserved canine semen.

## Results

### Characterization of the LDL nanoparticles

The physicochemical characteristics of three batches of the LDL nanoparticles are summarized in Table 1. The three preparation methods applied to the EYP influenced the mean diameter of the resulting LDL particles (the graph with measurement of the polydispersion index (PDI) and Zeta potential and then in the Supporting information). In addition, the PDI determined for LDL-P indicated a monodisperse system (PDI<0,3), while a polydisperse system was indicated by the PDI values obtained for the other systems. The zeta potential was determined to be negative [and above −5 mV] for all the formulations obtained.

**Table 1:** The means of the diameter, polydispersion index value, and zeta potential (ζ) value of the low-density lipoprotein nanoparticles produced by different techniques

Technique	EYP			LDL-B			LDL-P			LDL-H		
Parameter	Peak 1	Peak 2	Peak 3	Peak 1	Peak 2	Peak 3	Peak 1	Peak 2	Peak 3	Peak 1	Peak 2	Peak 3
Size ± SD d.nm)	363.7 ± 95.3	1134.7 ± 186.3	1485 ± 2539.8	328 ± 29.2	58.9 ± 4.4	3478.3 ± 3012.3	254 ± 47.2	32.4 ± 29.3	3062 ± 0.0	319.9 ± 63.8	51.6 ± 9.4	3636.3 ± 3149.1
% Intensity ± SD	75.3 ± 0.8	18.8 ± 5.9	5.8 ± 5.7	79.8 ± 0.7	19.1 ± 0.8	1.1 ± 1.2	92.1 ± 7.04	7.2 ± 6.8	1.2 ± 0.0	69.3 ± 6.2	29.6 ± 6.9	0.9 ± 0.8
z-average ± SD (d.nm)	185.9 ± 6.4			175.5 ± 2.6			162.4 ± 4.8			145.6 ± 8.7		
PDI±SD	0.5 ± 0.04^c^			0.4 ± 0.01^b^			0.3 ± 0.06^a^			0.6 ± 0.1^d^		
Potencial-ζ (mV)	−4.63 ± 0.20			-3.55 ± 2.34			−1.15 ± 0.41			−4.93 ± 0.25		

EYP, egg yolk plasma; LDL-B, ultrasound bath; LDL-P, ultrasound tip; LDL-H, high-pressure homogenizer; PDI, polydispersion index. Results are expressed as mean ± S.E.M. for the three replicates. Distinct letters (a–d) indicate significant difference within rows (*p* < 0.05).

### Effects of the LDL nanoparticles on the sperm kinetics of thawed canine semen samples

The total sperm motility of LDL-B and LDL-P was significantly higher than that of EYP and LDL-H (Fig. S1). Similarly, the progressive motility (PM) of LDL-P was higher (*p* < 0.05) than that of EYP and LDL-H (Fig. S1). Moreover, the LDL-H medium exhibited a significantly higher beat cross frequency (BCF) (*p* < 0.05) than the EYP control (Fig. S2). However, for LDL-B and LDL-P, the BCF value did not differ significantly compared to the different experimental groups.

Furthermore, the average trajectory distance, curvilinear distance, average trajectory velocity, and curvilinear velocity (Fig. S3) values were significantly higher for EYP than LDL-P and LDL-H (*p* < 0.05). On the other hand, progressive linear distance and progressive linear velocity did not differ significantly among EYP, LDL-B, and LDL-P (*p* > 0.05) (Fig. S3). The treatments did not present any significant differences among each other in the straightness, linearity, oscillation coefficient, and amplitude of lateral head displacement values.

### Effects of the LDL nanoparticles on sperm structure and sperm integrity in thawed canine semen samples

The nanoparticles from the different treatment systems presented significantly (*p* < 0.05) decreased levels of intracellular reactive oxygen species (ROS) formed during freezing (Fig. S4) compared to the EYP control. LDL-P presented greater integrity (Fig. S1) and lower sperm membrane fluidity (MF) than the EYP control (*p* < 0.05; Fig. S1). The other parameters (presented in Table 2) did not differ statistically.

**Table 2:** The means of the sperm cell integrity parameters for the canine semen samples frozen with the prepared LDL nanoparticles

Variables	EYP	LDL-B	LDL-P	LDL-H
Lipid peroxidation (%)	64.59 ± 3.98	71.31 ± 3.26	71.42 ± 3.09	68.34 ± 3.09
Cell disruption index	35.18 ± 2.47	38.44 ± 2.49	38.79 ± 2.75	35.24 ± 2.09
Acrosome integrity (%)	26.88 ± 2.50	32.30 ± 2.88	33.57 ± 3.16	30.48 ± 2.80
Mitochondrial membrane potential (%)	57.69 ± 3.75	59.72 ± 2.64	58.90 ± 3.63	65.27 ± 3.19
DNA fragmentation index	0.092 ± 0.015	0.094 ± 0.012	0.091 ± 0.010	0.089 ± 0.011

EYP, egg yolk plasma; LDL-B, ultrasound bath; LDL-P, ultrasound tip; LDL-H, high-pressure homogenizer. Means (S.E.M.) did not differ between groups (*p* > 0.05).

### Correlations

Cellular damage at the DNA level was highly correlated (r = 0.51) with cell disruption (*p* < 0.0001). The significant Pearson’s correlation coefficients between the LDL micelle dimensions and the post-thaw sperm kinetics parameters for the canine semen cryopreservation extenders are listed in Table 3.

**Table 3:** Significant Pearson’s correlation coefficients (*p* < 0.05) between the post-thaw sperm kinetics parameters and the LDL micelle dimensions for the canine semen cryopreservation extenders

Parameter	Diameter	MT
Head lateral displacement range	0.2848	0.2184
Average distance of trajectory	0.2357	−0.1826
curvilinear distance	0.2651	−0.2146
progressive linear distance	0.1884	−0.2176
Measuring velocity of trajectory	0.2631	−0.1333
curvilinear speed	0.2962	−0.1660
Progressive linear velocity	0.2155	−0.1740
Cross flagellar beat frequency	−0.1697	−0.4873∗

MT, Motility Total. Significant Pearson’s Coefficients. ∗*p* < 0.0001.

## Discussion

The results of the present study revealed that LDL-P offered greater cryoprotection than the EYP control diluent, as evidenced by the maintenance of total and progressive sperm motility and sperm membrane integrity (MI), along with the lower levels of sperm MF and formation of intracellular ROS than the control. This was attributed to the high-reactivity surface of the LDL nanoforms obtained after processing with the ultrasound probe. The best results obtained with LDL-P could be associated primarily with the PDI of this formulation which revealed this formulation to be a monodisperse and stable system, while the other experimental formulations were demonstrated to be polydisperse solutions. These findings proved that the ultrasound probe generator (LDL-P) technique was more efficient for the homogenization, emulsification, and dispersion of the LDL nanoparticles from EYP.

Sperm freezing and thawing may induce sperm capacitation (cryocapacitation), which is reflected by increased MF, hyperactivation, and changes in the motility patterns and flagellar beats (1, 2). In the present study, these changes were more evident in the kinetics of the EYP control samples, as evidenced by the high values of velocity, distance, and sperm kinetics obtained for these samples.

The enhancement of cryoprotection observed with the use of LDL-P micelles could be associated with the changes in the physicochemical properties of these micelles, such as changes in the diameter and surface electricity. These physicochemical properties are closely associated with the dynamics of the interface between the nanoparticles and the lipid bilayers, in addition to the dynamics of the solid–liquid interface, as well as with the changes occurring during the interaction of the particles with the suspension medium and biological molecules (3, 4), including the semen plasma proteins (5).

The sperm plasma membrane is the structure that is mainly injured during seminal manipulation (6). The fluidity of the plasma membrane is influenced by the temperature to which it is exposed. The storage temperature between 19 ° and 5 °C causes the membrane to move into a gel phase, increasing the loss of lipids that are part of the cell structure, causing a loss in membrane selectivity (7). The results of the present study revealed that LDL-B and LDL-P were more efficient in maintaining the sperm plasma MI than the other diluents, probably due to higher surface reactivity and a probable increase in the interaction capacity of the nanoforms due to production using ultrasonication. This ability to adhere to the sperm plasma membrane and protect it against heat shock (8, 9) is the key cryoprotective effect of LDL, along with the promotion of the entry of phospholipids and cholesterol into the cell membrane (9).

The exact mechanism of the factors associated with the interface between the nanoparticles and the cell membranes could be influenced by a large and complex set of interactions as these membranes are fluid thermodynamic structures with surface electrical irregularities (10, 11). Therefore, deciphering this mechanism would require complex studies. Furthermore, the secretion of products, such as ions and proteins, from the cells causes the interface to be dynamic in nature, thereby leading to the modification of the nanoparticles when they interact with the cells (12).

Cryopreservation may also damage the cytoskeleton and sperm mitochondria due to the formation of high levels of ROS, which destabilize all the cellular and fertilization processes (13). Among the different diluents used in the present study, EYP presented the highest levels of ROS. Cryocapacitation elevates the intracellular ROS levels, mainly due to increased flagellar and mitochondrial activity (14). Therefore, it could be inferred that EYP nanoprocessing produced LDL nanoforms with greater oxidizing capacity, which suppressed or eliminated the ROS formed during freezing (15). This effect could be attributed to the properties acquired during the nanoprocessing of LDL that increased the capacity for the adsorption of free ions, which is one of the properties explored upon the nanoprocessing of materials (16).

The high content of polyunsaturated fatty acids in the membrane and few antioxidant defenses render the sperms highly sensitive to oxidative stress (14, 17, 18). High levels of ROS elevate the lipid peroxidation, inactivate the glycolytic enzymes, and lead to sperm capacitation, and at extreme levels, to sperm deterioration (19, 20). This justifies the positive correlation among the ROS, LPO, and cell disruption.

The correlations revealed in the present study also demonstrated the relevance of analyzing the LDL micelles of the diluents. The greater the dimensions of these micelles, the greater is the indication of cryoinjuries, which are reflected as changes in MF, ROS, the kinetic indicators of sperm hyperactivation such as distance and velocities, and lower sperm MI and sperm motility. In addition, the PDI correlations demonstrated that the greater the differences in the particle diameter between the different diluents, the lower were the total and PM values of the sperms, probably due to the greater possibility of aggregation of the medium components and the unavailability of the particles for exerting their biological effect (21).

The present study reports a nanoprocessing applicable to LDL, which demonstrated positive results in terms of maintaining the sperm viability during semen freezing in various *in vitro* analyses. However, further investigations exploring the exact mechanism underlying the action of these nanoforms are warranted. In addition, the *in vivo* application of these nanoforms in artificial insemination should be investigated.

## Conclusion

Ultrasonication of EYP using a probe was demonstrated to be an efficient method to produce LDL nanoparticles for enhancing cryoprotection during canine semen freezing.

## Experimental procedures

### Preparation of the LDL nanoparticles

The LDL used in the present study was obtained from chicken eggs. The egg yolk was separated from egg white and placed on paper to remove the traces of egg white and the yolk membrane. In order to separate the EYP, 20 ml egg yolk was first diluted in 80 ml of Tris–glucose (Tris (hydroxymethyl)-aminomethane (3.025 g), sodium citrate monohydrate (1.7 g), fructose (1.25 g), benzylpenicillin (100 rag), dihydrostreptomycin sulfate (100 mg)) and distilled water (100 ml). The pH was 6.84 and osmolarity was 364 mOsm after addition of egg yolk (1). The mixture was then centrifuged at 10,000*g* for 45 min, followed by discarding the pellet and subjecting the supernatant to another two rounds of centrifugation at the same conditions. The supernatant from the third centrifugation was the EYP (2).

The obtained EYP was subsequently subjected to two ultrasound treatment systems—a bath-type generator (Eco-SONICS, Q3.0/40A, Ultronique) at 40 kHz for 30 min (LDL-B) and an ultrasound probe with a diameter of 3 mm (Bamdelin Electronic UW 2200) at 50% amplitude for 30 min (LDL-P). The temperature in the ultrasound treatment processes was controlled by immersing the diluent in a container with ice. In the third treatment system, the EYP was homogenized using Ultra-Turrax T10 (IKA) at 14,500 rpm for 2 min and then processed in a high-pressure homogenizer (Emulsiflex C3, Avestin) at 10,000 PSI for six cycles (LDL-H). Diluents from all treatment systems were stored at 5 °C for 7 days.

### Characterization of the LDL nanoparticles

The zeta potential and particle size values for the prepared LDL nanoparticles were determined by adopting the light scattering and laser-doppler methods, respectively, using Zeta Nano Series equipment (Malvern Instruments). In order to determine the zeta potential (ζ) of the nanoparticles, the samples were diluted in ultrapure water, and the mixture was placed in an electrophoretic cell with a potential of ±150 mV. The ζ-potential values were the mean values of the electrophoretic mobility calculated using the Smoluchowski equation.

The particle size measurements were performed at 25 °C at a detection angle of 90° after appropriately diluting the samples in distilled water. Each particle size measurement lasted for 300 s. The hydrodynamic radius was calculated using the Stokes–Einstein equation. All characterization experiments were performed in triplicate.

### Animals

The animal interventions used in the present study were approved by the Animal Experimentation Ethics Committee of the Faculty of Veterinary Medicine, Federal University of Pelotas (approval number: 9226/2014).

Ten clinically healthy male dogs (breeds: English bulldog, American bulldog, Australian cattle dog, and Golden retriever: n = 1 for each; Labrador retriever: n = 4; Shih Tzu: n = 2) aged 2 to 5 years, previously conditioned to semen collection, and with demonstrated fertility *in vivo* were used in the present study to obtain the semen samples.

Semen collection was performed weekly (2 fractions per dog). The second semen fraction, which is rich in sperm, was collected through digital manipulation (3). Only the samples exhibiting total motility greater than 80% were retained for further use (n = 20). Sperm concentration in the retained semen samples was determined using the Neubauer chamber. Each ejaculate was fractionated into four samples of equal volume. The four fractionated samples were diluted, respectively, with EYP, LDL-B, LDL-P, and LDL-H media, under isothermal conditions, thereby standardizing the sperm concentration to 200 × 10^6^ motile sperms/ml. After 1 h of incubation at 25 °C, the samples were diluted again in the diluents (at approximately 25 °C) containing glycerol at a final concentration of 5%, thereby achieving the sperm concentration of 100 × 106 motile sperms/ml.

In order to perform the cryopreservation process, the samples were poured into 0.25-ml straws, which were then placed inside a glass container, wrapped in paper, and refrigerated at 5 °C for 2 h. Afterward, the straws were placed at 5 cm in liquid nitrogen vapor for 10 min, followed by immersion in liquid nitrogen, and then storage for a minimum of 14 days. Thawing, when using the samples for analyses, was performed in a water bath at 37 °C for 30 s.

### Sperm motility

The parameters of sperm kinetics, namely, total motility, PM, curvilinear velocity, progressive linear velocity, average trajectory velocity, straightness, linearity, oscillation coefficient, flagellar cross beat frequency (BCF), the amplitude of lateral head displacement, curvilinear distance, progressive linear distance, and average trajectory distance, were measured. The measurements were performed under a phase-contrast microscope (Axio Scope A1, Zeiss) at 200× magnification, and the results were analyzed using an automated computer-assisted sperm analysis system (Sperm Vision 3.5, Minitube). The protocol began with diluting 50 μl of thawed semen in 150 μl of Tris–glucose, followed by incubation at 37 °C for 10 min. Afterward, a 2-μl drop of each sample was placed between a microscope glass slide and a coverslip prewarmed at 37 °C. The slides were then examined under a microscope across six randomly selected fields of observation, each containing 150 cells.

### Flow cytometry

The flow cytometric analysis was performed using an Acoustic Focusing Cytometer (Attune) equipped with blue (Argon 488 nm) and violet (UV 450 nm) lasers. The fluorescent probes were purchased from Invitrogen. The green, orange, and red fluorescence signals were detected using the photomultipliers BL1 (530/30 filter), BL2 (575/24 filter), and BL3 (>640 filter), respectively. The flower stability cytometer essence was evaluated daily using a standard solution (Invitrogen).

A total of 20,000 sperm events were examined per sample at a flow rate of 200 cells/s. In order to visualize the sperm population, the nonsperm events and cell debris were eliminated from the FSC × SSC scatter plots (4) through exposure to Hoescht 33342 (2 mM), followed by the detection of the fluorochrome using photomultiplier VL1 (filter 450/40); this process was not performed for the DNA fragmentation analysis. The results were obtained using the Attune Cytometric software V2.1.

### Sperm membrane fluidity

The sperm MF was evaluated using 1 μM of the YO-PRO marker and Merocyanine 540 (M540) at a final concentration of 2.7 μM. Live sperms (YO-PRO-negative sperm) were categorized into high fluidity (high M540 concentration) and low fluidity (low M540 concentration) cells (4).

### Plasma MI

MI of the sperm cells was assessed by performing the staining analysis using 20 μM carboxyfluorescein diacetate (CFDA) and 7.5 μM propidium iodide (PI). The sperm cells exhibiting MI were stained green due to cytoplasmic penetration and retention of CFDA, while red fluorescence due to propidium iodide staining was exhibited only by the sperm cells having membrane injuries and not the live sperm cells. Only the gametes labeled with CFDA alone were considered to exhibit MI (4)

### Mitochondrial membrane potential

Mitochondrial membrane potential of the sperm cells was assessed using the marker Rhodamine 123 (100 nM) in combination with PI (7.3 μM). Only the intact sperms (PI-negative sperms), which exhibited intense green fluorescence and high rhodamine accumulation, were considered to exhibit high mitochondrial functionality. On the other hand, the sperm cells exhibiting low-intensity green staining were considered to have low mitochondrial functionality (5).

### Acrosome integrity

The acrosomes (ACROs) were analyzed using the *Arachis hypogaea* lectin conjugated with fluorescein isothiocyanate (20 μl; 20 mg/ml) in combination with PI (20 μl). The intact ACROs displayed a crescent shape and emitted green fluorescence. On the other hand, the sperms with damaged ACROs did not display the normal morphology and emitted red fluorescence (6).

### DNA fragmentation index

The DNA fragmentation index was determined using the chromatin structure assay. The samples were exposed to TNE [0.01 M Tris–HCl, 0.15 M NaCl, and 0.001 M EDTA; pH 7.2] (Sigma) for 30 s and then to Triton (Triton X-1, 0.1%; Sigma). This step was immediately followed by the addition of acridine orange (2 mg ml–1) as a marker. This marker emits green fluorescence when intercalated within the dsDNA helix and red fluorescence when it associates with denatured ssDNA (4).

### Production of intracellular ROS

The production of intracellular ROS was determined for all samples using an oxidative stress marker named carboxy-H2DCF-DA (1 mM) along with PI (7.5 μM). The measurements were undertaken at two different time points: first, immediately after the addition of fluorochromes to the semen sample and then after 60 min of incubation at 37 °C. ROS determination was based on the median intensity of green fluorescence (an indicator of oxidation) emitted by the viable sperms. The results were expressed as the intracellular ROS rate (4).

### Lipid peroxidation

The level of LPO was determined using the fluorophore BODYPY (5 μM), which emits red fluorescence in the absence of oxidation and green fluorescence in the presence of LPO. The evaluation was performed after 120 min of incubation of the sample with the fluorophore at 37 °C. The results were expressed as the percentage of sperms exhibiting LPO (7).

### Statistical analysis

The statistical analyses were performed using the Statistix 10 software (Analytical Software, 2014). The Shapiro–Wilk test was employed to verify the non-normality of the data. After ANOVA, the means were compared using the least significant difference test. The correlations were determined based on Pearson’s correlation coefficients. Significance was assigned to all means with *p* < 0.05.

## Data availability

No applicable.

## Supporting information

This article contains supporting information.

## Conflicts of interest

The authors declare that they do not have any commercial or associative interest that represents a conflict of interest in connection with the work submitted.
