# Supplementary material for: Probe ultrasonification of egg yolk plasma forms low-density lipoprotein nanoparticles that efficiently protect canine semen during cryofreezing
Source: J Biol Chem. 2022 Apr 28;298(7):101975. doi: 10.1016/j.jbc.2022.101975 (PMC9293657; doi:10.1016/j.jbc.2022.101975)
Supplement: Supplemental Figure S1 [file mmc1.docx]

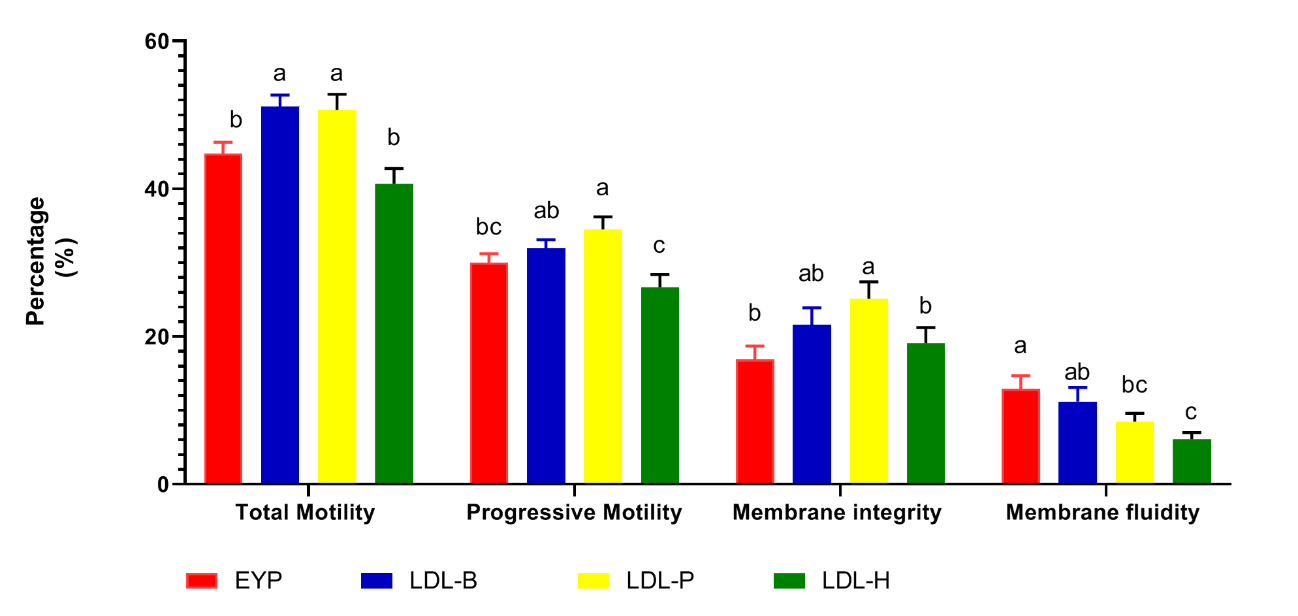


Figure 1. Total and progressive motility, membrane integrity and fluidity of canine semen cryopreserved with the different diluents (mean ± S.P.E.).

EYP, egg yolk plasma (red); LDL-B, ultrasound bath (blue); LDL-P, ultrasound tip (yellow); LDL-H, high pressure homogenizer (green). Different letters (a-c; A-B) above the bars indicate significant difference between groups (P < 0.05), (n= 20).
