# Supplementary material for: Probe ultrasonification of egg yolk plasma forms low-density lipoprotein nanoparticles that efficiently protect canine semen during cryofreezing
Source: J Biol Chem. 2022 Apr 28;298(7):101975. doi: 10.1016/j.jbc.2022.101975 (PMC9293657; doi:10.1016/j.jbc.2022.101975)
Supplement: Supplemental Figure S2 [file mmc2.docx]

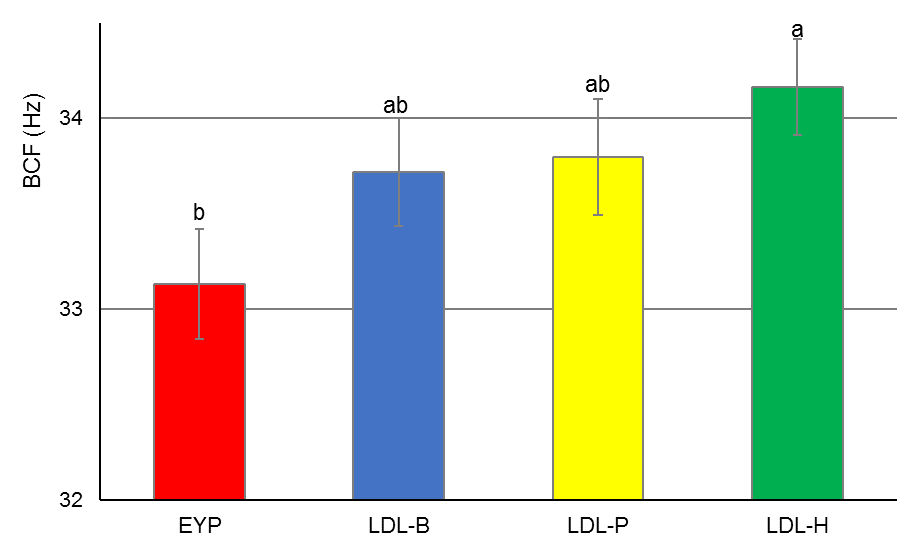


Fig 2. Averages (± E.P.M.) of flagellar cross beat frequency (BCF) for canine semen cryopreserved with the different diluents.

EYP, egg yolk plasma; LDL-B, ultrasound bath; LDL-P, ultrasound tip; LDL-H, high pressure homogenizer. Different letters (a-c) indicate significant difference between groups (P < 0.05), (n= 20).
