# Supplementary material for: Probe ultrasonification of egg yolk plasma forms low-density lipoprotein nanoparticles that efficiently protect canine semen during cryofreezing
Source: J Biol Chem. 2022 Apr 28;298(7):101975. doi: 10.1016/j.jbc.2022.101975 (PMC9293657; doi:10.1016/j.jbc.2022.101975)
Supplement: Supplemental Figure S3 [file mmc3.docx]

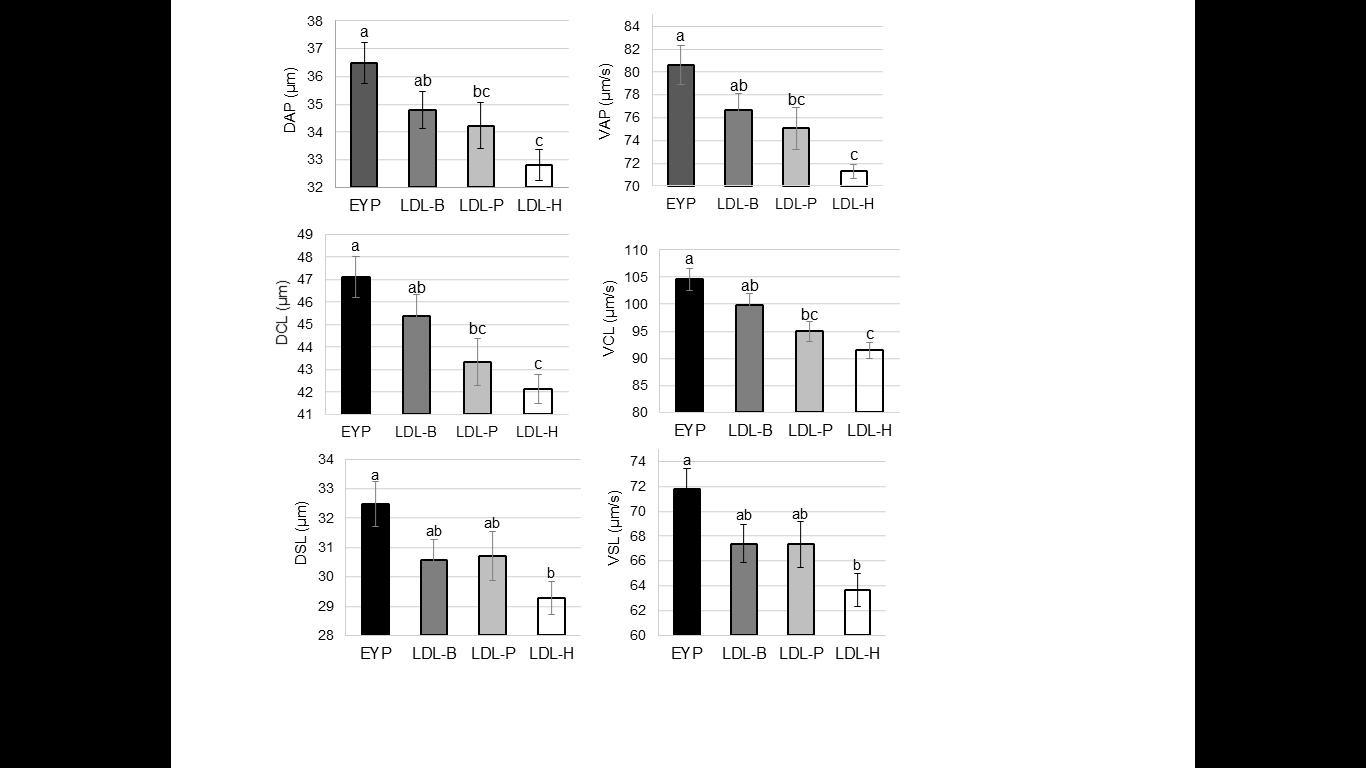


**Fig 3.** Sperm distance and velocity measurements after thawing with different LDL nanoforms (mean ± P.E.M).

DAP, Mean path distance; DCL, curvilinear distance; DSL, progressive linear distance; VAP, mean path velocity; VCL, curvilinear velocity; VSL progressive linear velocity; EYP, egg yolk plasma; LDL-B, ultrasound bath; LDL-P, ultrasound tip; LDL-H, high-pressure homogenizer. Different letters (a-c) indicate significant difference (P < 0.05), (n= 20).
